# Allogeneic red blood cell transfusion is an independent risk factor for 1-year mortality in elderly patients undergoing femoral neck fracture surgery: Retrospective study

**DOI:** 10.1097/MD.0000000000021897

**Published:** 2020-08-28

**Authors:** Hyeon Ju Shin, Jong Hun Kim, Seung-Beom Han, Jong Hoon Park, Woo Young Jang

**Affiliations:** a Department of Anesthesiology and Pain Medicine, Korea University College of Medicine; b Korea University Bloodless Medicine Center; c Division of Infectious Diseases, Department of Internal Medicine; d Department of Orthopedic Surgery, Korea University College of Medicine, Seoul, Republic of Korea.

**Keywords:** allogeneic red blood cell transfusion, elderly patients, hip fracture, chronic kidney disease, mortality

## Abstract

Allogeneic red blood cell transfusion (ABT) is 1 of the poor prognostic factors for morbidity and mortality in patients with hip fracture, particularly among elderly patients. This study aimed to investigate the risk factors for ABT and 1-year mortality in elderly patients undergoing surgery for femoral neck fracture.

A total of 225 elderly patients who underwent femoral neck fracture surgery between May 2013 and November 2015 at a tertiary medical center were retrospectively recruited. Medical records were analyzed.

The median patient age was 80 years and 28.4% were men. A total of 113 patients received ABT (50.2%). Multivariate logistic regression analysis showed that female sex (odds ratio [OR] 2.606, 95% confidence interval [CI] 1.283–5.295, *P* = .008), malignancy (OR 5.098, 95% CI 1.725–15.061, *P* = .003), chronic kidney disease stage ≥ 3 (OR 3.258, 95% CI 1.603–6.622, *P* = .001), and anemia (hemoglobin < 12 g/dL) (OR 4.684, 95% CI 2.230–9.837, *P* < .001) were significantly associated with ABT. The 1-year mortality rate after surgery was 15.1%. Male sex (OR 2.477, 95% CI 1.101–5.575, *P* = .028), ABT (OR 2.367, 95% CI 1.036–5.410, *P* = .041), and intensive care unit admission (OR 5.564, 95% CI 1.457–21.249, *P* = .012) were significantly associated with 1-year mortality.

In this study, underlying comorbidities such as chronic kidney disease and malignancy were associated with ABT. Furthermore, ABT was a significant independent risk factor for 1-year mortality. These findings suggest that underlying comorbidities and the need for ABT should be considered in the risk assessment of elderly patients with femoral neck fracture to improve the outcomes after surgery.

## 1 Introduction

Hip fracture in elderly patients is a serious health concern owing to the associated morbidity and mortality.^[1–3]^ The mortality at 1 month after hip fracture surgery approaches 10%. One of the reasons for the high mortality after hip fracture treatment is that patients with hip fracture already have many underlying diseases such as cardiovascular disease, kidney dysfunction, diabetes mellitus, and coagulation disorders.^[4,5]^ Anemia is also a common problem in elderly hip fracture patients with underlying diseases.^[6]^ Furthermore, blood loss occurs as a consequence of both the fracture and the surgery; thus, allogeneic red blood cell transfusion (ABT) is frequently needed to correct the anemia that results from fracture- or surgery-related blood loss. For these reasons, more than 20% of the patients with hip fracture may need to receive ABT.^[7,8]^

However, many authors have suggested that the use of ABT can increase the rate of infection after surgery in both elective and trauma orthopedic procedures.^[6,9]^ A systematic review also showed that ABT is associated with increased morbidity and mortality in critically ill patients, including trauma patients.^[10]^ Although ABT is 1 of the poor prognostic factors for morbidity and mortality in patients with hip fracture, the literature on the impact of ABT on 1-year mortality in elderly patients undergoing femoral neck fracture surgery is insufficient. Furthermore, it is not clear which predisposing factors may be associated with an increased risk of ABT. Therefore, the purpose of this study was to investigate the risk factors for ABT by using a prediction model and the 1-year mortality analysis in elderly patients undergoing surgery for femoral neck fracture.

## 2 Methods

This study was approved by the institutional review board at our institute (approval number: 2019AN0520). Elderly patients (≥ 64 years old) who were diagnosed with femoral neck fracture between May 2013 and November 2015 were retrospectively reviewed. Inclusion criteria included the following conditions;

(1) hospitalized patient ≥ 64 years,
(2) a patient who underwent surgery for femoral neck fracture.

Exclusion criteria included the following conditions;

(1) a patient who received iron or eythropoietin prior to the femoral neck fracture surgery.
(2) a patient who received iron or eythropoietin during the perioperative period.

Clinical variables, including demographic data, underlying medical comorbidities, and medication history, were collected. Preoperative clinical conditions including American Society of Anesthesiologists (ASA) physical status classification,^[11]^ complete blood count, and prothrombin time/international normalized ratio were recorded. Administration of ABT during the perioperative period was recorded. Perioperative intensive care unit (ICU) admission and 1-year mortality data were collected and analyzed.

Underlying medical comorbidities were defined as diabetes mellitus, pulmonary disease (including asthma and chronic obstructive pulmonary disease), malignancy (including solid organ cancer and hematologic malignancy), dementia, chronic kidney disease (CKD) stage ≥ 3 (glomerular filtration rate < 50 mL/min/1.73 m^2^), cerebrovascular disease (including hemorrhagic and ischemic stroke), hypertension (based on the criteria by the American Heart Association^[12]^), ischemic heart disease, and history of percutaneous coronary intervention. Medication history included

(1) hypertension or heart medications such as calcium channel blockers, angiotensin-converting enzyme inhibitors, angiotensin II receptor blockers, and beta-blockers, as well as
(2) anticoagulants such as direct oral anticoagulants, low molecular heparin, and warfarin.

The criteria of perioperative ICU admission were any critical surgical or medical conditions that would lead to potential or established organ failure, including respiratory failure, cardiac arrest, hemorrhagic shock, septic shock, and sudden fall in level of consciousness or prolonged seizures.

The indications for perioperative ABT were as follows:

(1) preoperative hemoglobin (Hb) < 7 g/dL for all patients or preoperative Hb < 8 g/dL for patients with a history of cardiovascular disease, and
(2) acute bleeding event during the perioperative period at the discretion of the treating physician.

### 2.1 Statistical analysis

Comparison analyses of clinical variables between 2 groups of patients (with and without perioperative ABT) were performed. Dichotomous variables were compared using the Pearson *χ*^2^ test or Fisher exact test. For continuous variables, the Mann-Whitney test was used. The Wilcoxon signed-rank test was used for ABT prediction scores between patients with ABT and those without ABT. The sensitivity and specificity of the ABT prediction model were calculated for each score value. The performance of the ABT prediction model was evaluated using receiver operating characteristic (ROC) curves^[13]^ with calculation of the area under the ROC curve. Variables with a *P* value of < .1 on the comparison analysis were included in a multiple logistic regression analysis to determine the risk factors associated with ABT. Odds ratios (ORs) and 95% confidence intervals (CIs) were calculated. A value of *P* < .05 was considered statistically significant. Using the above-mentioned method, comparison analyses between patients with 1-year mortality and those without 1-year mortality, as well as subsequent multiple regression analysis were performed.

## 3 Results

During the study period, there was no use of intraoperative cell salvage with autologous transfusion for femoral neck fracture surgery at our institution. A total of 225 patients were enrolled. The patient cohort included 64 males (28.4%) and 161 females (71.6%). The median patient age was 80 years with an interquartile range of 75 to 86 years. The common underlying medical comorbidities included hypertension (72.4%), diabetes mellitus (33.3%), CKD stage ≥ 3 (27.6%), cerebrovascular disease (22.7%), and ischemic heart disease (22.2%). Perioperative ABTs were performed in 113 patients (50.2%) (median of 2 units of transfusion interquartile range 2–3 units]), and the 1-year mortality rate was 15.1%.

**Figure 1:**
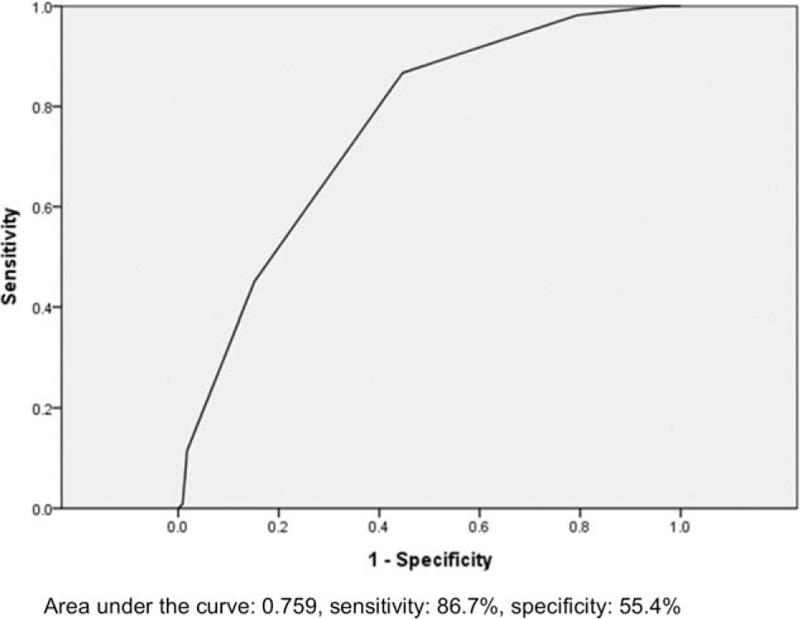
Receiver operating characteristic curve for the allogeneic red blood cell transfusion prediction model.

Patients were categorized into 2 groups based on perioperative ABT (perioperative ABT group, n = 113 [50.2%] and non-perioperative ABT group, n = 112 [49.8%]) to determine factors associated with perioperative ABT. The perioperative ABT group included significantly more patients with older age ≥75 years (85.0% vs 71.4%, *P* = .014), female sex (78.8% vs 64.3%, *P* = .016), malignancy (15.9% vs 5.4%, *P* = .010), and CKD stage ≥3 (38.9% vs 16.1%, *P* < .001) than the non-perioperative ABT group. With respect to preoperative clinical conditions, there were more patients with low preoperative Hb < 12 g/dL (87.6% vs 58.0%, *P* < .001) and ASA 3 (37.2% vs 23.2%, *P* = .023) in the perioperative ABT group (Table 1). Each of the 6 significant variables (older age ≥75 years, female sex, malignancy, CKD stage ≥ 3, low preoperative Hb < 12 g/dL, and ASA 3) identified from the comparison analysis was given a point score for the creation of the perioperative ABT prediction model. Patients with perioperative ABT had higher scores when tested using the Wilcoxon signed-rank test (*P* < .001) (Table 2). The ROC curve had an area under the curve of 0.759 for the perioperative ABT prediction model (Fig. 1). The ideal threshold score of 3 was identified from the ROC curve with a sensitivity of 86.7% and a specificity of 55.4%. In a multivariate logistic regression analysis using variables with *P* < .1 from the comparison analysis, female sex (OR 2.606, 95% CI 1.283–5.295, *P* = .008), malignancy (OR 5.098, 95% CI 1.725–15.061, *P* = .003), CKD stage ≥ 3 (OR 3.258, 95% CI 1.603–6.622, *P* = .001), and low preoperative Hb < 12 g/dL (OR 4.684, 95% CI 2.230–9.837, *P* < .001) were significantly associated with perioperative ABT. This analysis revealed that older age ≥ 75 years (OR 2.008, 95% CI 0.939–4.297, *P* = .072) and ASA 3 (OR 1.907. 95% CI 0.963–3.777, *P* = .064) had a borderline association.

**Table 1:**
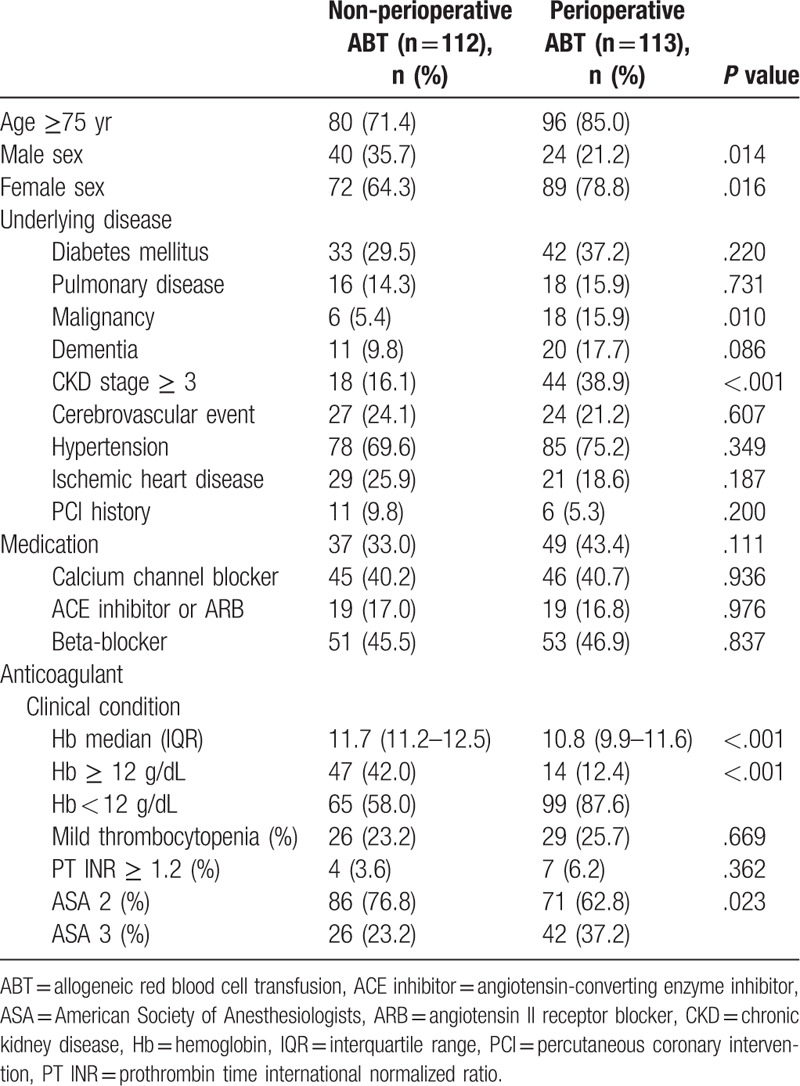
Characteristics of elderly patients with femoral neck fracture who underwent orthopedic surgery stratified according to administration of perioperative allogeneic red blood cell transfusion.

**Table 2:**
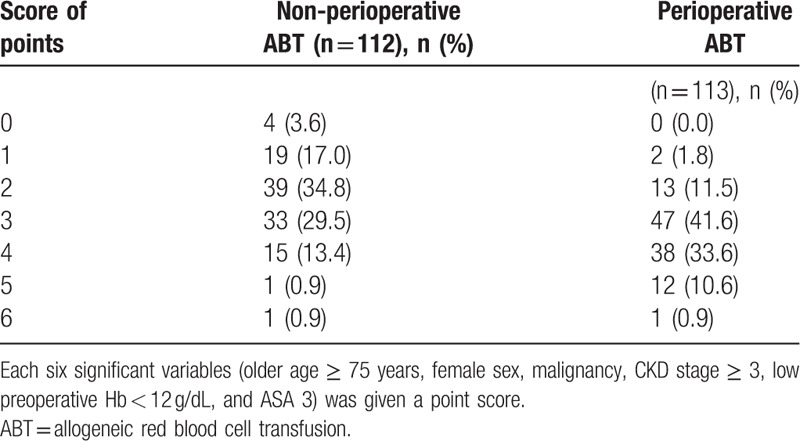
Perioperative allogeneic red blood cell transfusion prediction score.

The rate of 1-year mortality after femoral neck fracture surgery was 15.1%. The patients were categorized in 2 groups (survival at 1 year, n = 191 [84.9%] and non-survival at 1 year, n = 34 [15.1%]). For the non-survival group, there were significantly more patients with older age ≥ 75 years (91.2% vs 75.9%, *P* = .047), international normalized ratio ≥ 1.2 (14.7% vs 3.1%, *P* = .014), ASA 3 (47.1% vs 27.2%, *P* = .020), perioperative ABT (67.6% vs 47.1%, *P* = .027), and perioperative ICU admission (14.7% vs 2.6%, *P* = .009) when compared with the survival group. Higher proportions of male sex (41.2% vs 26.2%, *P* = .074), CKD stage ≥ 3 (41.2% vs 25.1%, *P* = .054), and low preoperative Hb < 12 g/dL (85.3% vs 70.7%, *P* = .077) with a borderline significance were noted in the non-survival group (Table 3). In a multivariate logistic regression analysis using variables with *P* < .1 from the comparison analysis, male sex (OR 2.477, 95% CI 1.101–5.575, *P* = .028), perioperative ABT (OR 2.367, 95% CI 1.036–5.410, *P* = .041), and perioperative ICU admission (OR 5.564, 95% CI 1.457–21.249, *P* = .012) were significantly associated with 1-year mortality.

**Table 3:**
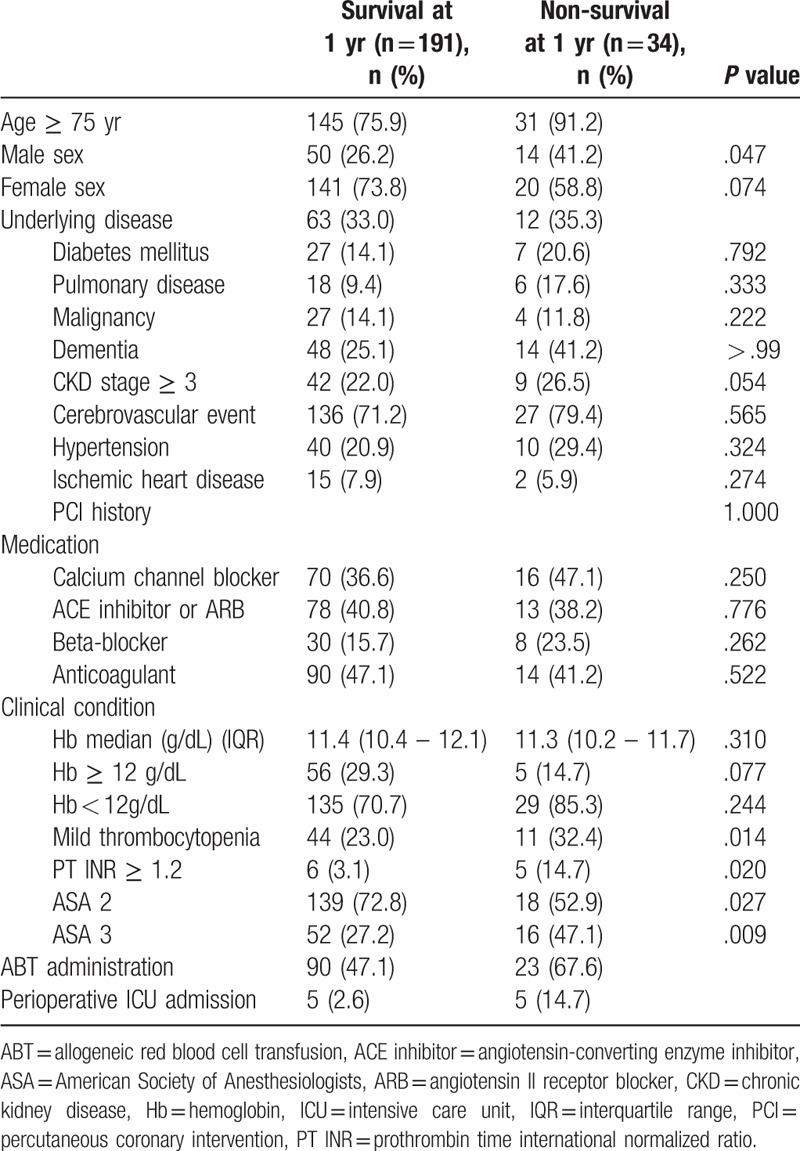
Characteristics of elderly patients with femoral neck fracture who underwent orthopedic surgery stratified according to survival and non-survival at 1 year.

## 4 Discussion

ABT is a well-known poor prognostic factor for morbidity and mortality in patients undergoing orthopedic surgery.^[9,14]^ Therefore, to reduce the rate of ABT, it is important to identify predisposing factors, especially in elderly hip fracture patients who are at risk of high morbidity and mortality. In this study, female sex, a history of malignancy, CKD stage ≥ 3, low preoperative Hb, ASA > 3, and older age ≥ 75 years were significant risk factors for ABT. On the basis of these findings, a perioperative ABT prediction model was created, which would be useful for health-care providers in evaluating the probability of perioperative ABT in risk assessment for perioperative management. In this study, ABT was a significant independent risk factor for 1-year mortality after hip fracture surgery.

Multivariate logistic regression analysis identified six factors in this study, consistent with previous reports.^[3–5,15,16]^ Older age ≥ 75 years has been consistently reported as a risk factor for transfusion in several studies on hip fracture,^[8,16]^ possibly owing to the decreased hematopoietic activity, decreased platelet function, and reduced physician threshold for transfusion in these patients.^[17]^ Although the elderly population was considered to include individuals older than 65 years in recent studies,^[18,19]^ the accepted definition of the elderly population has been changed to individuals 75 years and older owing to the delay in aging resulting from advancements in medicine.^[16]^ It has been reported that age > 75 years is a risk factor for the need for blood transfusions and is the primary risk factor for 1-year mortality in patients with hip fractures. Corresponding with earlier reports,^[8,16]^ our study showed that older age (≥ 75 years) had a borderline association with ABT. Female sex is also a well-known risk factor for ABT after a major orthopedic operation,^[20]^ which may be attributed to the smaller body size, including body surface area, body mass index, or body weight, of women.^[21]^ Therefore, surgeons should make efforts to minimize blood loss during surgery, especially in elderly female patients with hip fracture.

Preoperative anemia and CKD stage ≥ 3 have been identified as additional risk factors,^[22]^ and these findings were confirmed in the present study. The rate of ABT among CKD patients aged ≥ 65 years is estimated at 11 to 14 per 100 person-years.^[23]^ CKD is also associated with comorbidities such as obesity, diabetes mellitus, and hypertension, each of which is an independent risk factor for complications after surgery.^[24]^ Hsiue et al.^[25]^ reported that CKD patients have worse outcomes after surgery for hip fracture than patients without kidney disease. Furthermore, acute kidney injury can cause high mortality after an operation for hip fracture.^[5,15,26]^ Therefore, there is a need for multidisciplinary clinical pathways to maintain kidney function by minimizing the use of nephrotoxic drugs and providing appropriate fluid management. Especially, with respect to blood management, use of erythropoiesis-stimulating agents together with iron therapy should be considered in patients with CKD.^[27,28]^ Erythropoietin (EPO), which is mainly secreted by the adult kidney, is an essential regulator of red blood cell production.^[29]^ Therefore, in patients with CKD, iron therapy without EPO may not be effective. On the other hand, iron supplementation with EPO can be helpful in treating anemic patients with CKD.^[30,31]^ Furthermore, a meta-analysis^[32]^ reported that preoperative EPO administration can reduce the rate of ABT in patients undergoing total hip arthroplasty or total knee arthroplasty.

ASA > 3 was revealed to be a borderline significant risk factor for ABT. Although ABT is prevalent in chronically ill patients, the criteria for optimal anemia management are not clearly defined. Furthermore, the use of ABT for the treatment of anemia in chronically ill patients warrants further evaluation because a previous randomized controlled trial suggested that the liberal use of transfusions may have resulted in higher hospital mortality rates.^[33]^ The presence of a history of malignancy was a significant risk factor for ABT in this study. According to a previous report, solid neoplastic diseases frequently occur in the elderly. It is well known that such diseases induce anemia and that their specific treatments have myelotoxic effects. One of the most important findings of this study was that the ABT prediction model showed that if a patient had 3 concurrent predisposing risk factors out of the 6 risk factors, the ABT prediction score had a 86.7% sensitivity and 55.4% specificity. Our prediction model might be applied to differentiate patients at a higher risk for ABT from those with a low risk during femoral neck fracture surgery, to better plan the perioperative management and improve outcomes.

In this study, ABT was a significant independent risk factor for 1-year mortality after hip fracture surgery. Many previous studies have reported that the mortality rate within the first year after proximal femoral fracture was increased to between 21.9% and 35.7%.^[2–4]^ In this study, the overall mortality rate was 15.1%; however, the rate was increased to 22% among patients with ABT. ABT can cause infectious disease transmission, systemic and local immune modulation, cardiac overload, lung injury, and matching errors.^[10]^ Furthermore, fluid overload is a common complication after ABT in patients undergoing hip surgery and ABT-associated circulatory overload is the second leading cause of ABT-related death.^[34]^ Therefore, physicians should attempt to administer anemia-correcting agents instead of ABT to correct low preoperative Hb level and should perform cautious fluid management. For this reason, blood management using iron supplements and/or EPO and/or anti-fibrinolytics should be considered when planning the treatment strategy for hip fracture patients with several risk factors for ABT.^[30,32,35]^

This study had several limitations. First, this was retrospective study, which makes it prone to misinterpretations. Therefore, further prospective studies with a larger sample size are necessary to generalize the results of this study. Second, although the current ABT guidelines are mostly based on arbitrary Hb triggers, the Hb level when administering ABT might differ according to physiological parameters and clinical variables. Third, effects from acute physiologic events, such as infection or sequential organ failure and ICU admission caused by hemorrhagic shock or other processes, were not analyzed, and this limitation might have affected our analysis of 1-year mortality. Therefore, further studies are urgently needed to elucidate these important confounders.

## 5 Conclusion

Underlying comorbidities such as CKD and malignancy were associated with ABT in this study. Furthermore, ABT was a significant independent risk factor for 1-year mortality. These findings suggest that underlying comorbidities and the need for ABT should be considered in the risk assessment of elderly patients with hip fracture to improve the outcomes after surgery.

## Author contributions

**Conceptualization:** Hyeon Ju Shin, Jong Hun Kim, Seung-Beom Han, Jong-Hoon Park, Woo Young Jang.

**Data curation:** Hyeon Ju Shin, Jong Hun Kim.

**Formal analysis:** Seung-Beom Han, Jong-Hoon Park.

**Supervision:** Jong-Hoon Park.

**Writing – original draft:** Hyeon Ju Shin, Jong Hun Kim.

**Writing – review & editing:** Woo Young Jang.
